# Correction to: Ravulizumab: A Review in Atypical Haemolytic Uraemic Syndrome

**DOI:** 10.1007/s40265-021-01517-x

**Published:** 2021-04-19

**Authors:** Yahiya Y. Syed

**Affiliations:** Springer Nature, Private Bag 65901, Mairangi Bay, Auckland, 0754 New Zealand

## Correction to: Drugs 10.1007/s40265-021-01481-6

The article Ravulizumab: A Review in Atypical Haemolytic Uraemic Syndrome, written by Yahiya Y. Syed, was originally published electronically in SpringerLink on 18 March 2021 without open access. After publication online Alexion Pharmaceuticals, Inc. Boston, MA, requested that the article be Open Choice to make the article an open access publication. Post-publication open access was funded by Alexion Pharmaceuticals, Inc. Boston, MA. This article is licensed under a Creative Commons Attribution-NonCommercial 4.0 International License, which permits any non-commercial use, sharing, adaptation, distribution and reproduction in any medium or format, as long as you give appropriate credit to the original author(s) and the source, provide a link to the Creative Commons licence, and indicate if changes were made. The images or other third party material in this article are included in the article's Creative Commons licence, unless indicated otherwise in a credit line to the material. If material is not included in the article's Creative Commons licence and your intended use is not permitted by statutory regulation or exceeds the permitted use, you will need to obtain permission directly from the copyright holder. To view a copy of this licence, visit http://creativecommons.org/licenses/by-nc/4.0/.

The original article has been corrected.

